# Breastfeeding Is Associated With a Reduced Maternal Cardiovascular Risk: Systematic Review and Meta‐Analysis Involving Data From 8 Studies and 1 192 700 Parous Women

**DOI:** 10.1161/JAHA.121.022746

**Published:** 2022-01-11

**Authors:** Lena Tschiderer, Lisa Seekircher, Setor K. Kunutsor, Sanne A. E. Peters, Linda M. O’Keeffe, Peter Willeit

**Affiliations:** ^1^ Clinical Epidemiology Team Medical University of Innsbruck Innsbruck Austria; ^2^ National Institute for Health Research Bristol Biomedical Research Centre University Hospitals Bristol and Weston National Health Service Foundation Trust and the University of Bristol Bristol United Kingdom; ^3^ Translational Health Sciences Bristol Medical School University of Bristol Learning & Research Building (Level 1) Southmead Hospital Bristol United Kingdom; ^4^ The George Institute for Global Health School of Public Health Imperial College London London United Kingdom; ^5^ Julius Center for Health Sciences and Primary Care University Medical Center Utrecht Utrecht the Netherlands; ^6^ The George Institute for Global Health University of New South Wales Sydney New South Wales Australia; ^7^ School of Public Health, Western Gateway Building University College Cork Cork Ireland; ^8^ MRC Integrative Epidemiology Unit University of Bristol United Kingdom; ^9^ Population Health Sciences Bristol Medical School University of Bristol United Kingdom; ^10^ Department of Public Health and Primary Care University of Cambridge United Kingdom

**Keywords:** breastfeeding, cardiovascular diseases, maternal risk, meta‐analysis, systematic review, Cardiovascular Disease, Epidemiology, Pregnancy

## Abstract

**Background:**

Breastfeeding has been robustly linked to reduced maternal risk of breast cancer, ovarian cancer, and type 2 diabetes. We herein systematically reviewed the published evidence on the association of breastfeeding with maternal risk of cardiovascular disease (CVD) outcomes.

**Methods and Results:**

Our systematic search of PubMed and Web of Science of articles published up to April 16, 2021, identified 8 relevant prospective studies involving 1 192 700 parous women (weighted mean age: 51.3 years at study entry, 24.6 years at first birth; weighted mean number of births: 2.3). A total of 982 566 women (82%) reported having ever breastfed (weighted mean lifetime duration of breastfeeding: 15.6 months). During a weighted median follow‐up of 10.3 years, 54 226 CVD, 26 913 coronary heart disease, 30 843 stroke, and 10 766 fatal CVD events were recorded. In a random‐effects meta‐analysis, the pooled multivariable‐adjusted hazard ratios comparing parous women who ever breastfed to those who never breastfed were 0.89 for CVD (95% CI, 0.83–0.95; I^2^=79.4%), 0.86 for coronary heart disease (95% CI, 0.78–0.95; I^2^=79.7%), 0.88 for stroke (95% CI, 0.79–0.99; I^2^=79.6%), and 0.83 for fatal CVD (95% CI, 0.76–0.92; I^2^=47.7%). The quality of the evidence assessed with the Grading of Recommendations Assessment, Development, and Evaluation tool ranged from very low to moderate, which was mainly driven by high between‐studies heterogeneity. Strengths of associations did not differ by mean age at study entry, median follow‐up duration, mean parity, level of adjustment, study quality, or geographical region. A progressive risk reduction of all CVD outcomes with lifetime durations of breastfeeding from 0 up to 12 months was found, with some uncertainty about shapes of associations for longer durations.

**Conclusions:**

Breastfeeding was associated with reduced maternal risk of CVD outcomes.

**Clinical Perspective**

**What Is New?**

- In our systematic review and meta‐analysis of 8 studies and >1 million parous women, we found that women who breastfed had a lower risk of future cardiovascular disease, coronary heart disease, stroke, and fatal cardiovascular disease.
- We found a progressive reduction of cardiovascular risk with lifetime durations of breastfeeding of up to 12 months.

**What Are the Clinical Implications?**

- Positive effects of breastfeeding on mothers need to be communicated effectively, awareness for breastfeeding recommendations needs to be raised, and interventions to promote and facilitate breastfeeding need to be implemented and reinforced.

The World Health Organization recommends to breastfeed children after birth.^1^ Specifically, children should be exclusively breastfed for the first 6 months of life and further breastfed for up to 2 years or beyond accompanied by complementary feeding.^1^ Despite these recommendations, a recent meta‐analysis using data from nationally representative surveys of countries from all over the world reported that only 37% of children <6 months of age are exclusively breastfed in low‐income and middle‐income countries.^2^ Furthermore, the prevalence of breastfeeding at 12 months was <20% in most high‐income countries.^2^

A key rationale for the World Health Organization recommendation is that breastfeeding has well‐established benefits for the child.^2^ For instance, previous studies demonstrated that children who have been breastfed are less likely to die from infectious diseases^3^ or experience respiratory infections.^4^ Besides beneficial effects on the child’s health, breastfeeding may also impact risk for several diseases in the mother.^2^ For instance, large‐scale meta‐analyses showed robust associations of breastfeeding with a reduced maternal risk of breast and ovarian carcinoma^5^ as well as type 2 diabetes.^6^

Emerging evidence from multiple individual studies suggests that lactation could also be associated with reduced cardiovascular risk in the later life of breastfeeding mothers and that the inverse association may be strengthened with longer durations of breastfeeding.^7^, ^8^, ^9^, ^10^, ^11^, ^12^, ^13^, ^14^, ^15^, ^16^, ^17^, ^18^, ^19^, ^20^ The 2021 Scientific Statement of the American Heart Association on Cardiovascular Disease Prevention in Women concluded that “lactation and breastfeeding may lower a woman’s later cardiometabolic risk,” although there exist some inconsistencies about the strength of the association and dose–response relationships.^21^ A recent umbrella review highlighted this inverse relationship in a narrative summary but did not conduct a formal meta‐analysis to obtain a pooled estimate for the association between breastfeeding and cardiovascular risk.^22^ To clarify these uncertainties, we conducted a systematic literature review and meta‐analysis with the aim of precisely characterizing the association between breastfeeding and development of maternal cardiovascular events.

## Methods

The current analysis was conducted according to the guidelines in the Preferred Reporting Items for Systematic Reviews and Meta‐Analyses statement (for checklist, see Table S1). P.W. is the guarantor of this work and, as such, had full access to all data in the study and takes responsibility for the integrity of the data and the accuracy of the data analysis. The data that support the findings of this study are available from the corresponding author upon reasonable request.

We screened the literature databases PubMed and Web of Science to find studies eligible for inclusion into the present analyses. Prospective studies were included in the review if they evaluated the association between history of breastfeeding and incidence of cardiovascular events in parous women. We used the search terms (“breastfeeding” OR “lactation”) AND (“cardiovascular disease” OR “stroke” OR “coronary heart disease” OR “coronary artery disease” OR “myocardial infarction”) in PubMed and TS=((“breastfeeding” OR “lactation”) AND (“cardiovascular disease” OR “stroke” OR “coronary heart disease” OR “coronary artery disease” OR “myocardial infarction”)) in Web of Science and searched for studies published until April 16, 2021, without language restrictions. In addition, we screened reference lists of relevant articles to identify additional relevant records.

A total of 2 independent reviewers (L.T. and L.S.) screened the identified literature and selected eligible studies. Data on the following variables were extracted from the publications and cross‐checked between the reviewers: study name, study design, country, year of baseline, total number of participants, number of parous women, inclusion of women with stillbirths, information on history of cardiovascular disease (CVD), study exclusion criteria, baseline age, reproductive factors (including parity, age at first child, age at last child, age at menarche, age at menopause, percentage of individuals with menopause, lifetime duration of breastfeeding, and number of individuals who ever/never breastfed), definitions of cardiovascular outcomes, duration of follow‐up, level of adjustment (o=adjusted for demographics and reproductive factors; +=adjusted for demographics and cardiovascular risk factors; ++=adjusted for demographics, reproductive factors, and cardiovascular risk factors), number of cardiovascular outcomes, hazard ratios, and 95% CIs. Inconsistencies were discussed and resolved by mutual agreement. In case we identified multiple publications of studies using data of the same cohorts, we only included multiple publications into our analyses if they reported effect sizes for different cardiovascular outcomes (eg, coronary heart disease [CHD] or stroke). For our analyses, cardiovascular events were classified into the following 4 categories: (1) fatal and/or nonfatal CVD, (2) fatal and/or nonfatal CHD, (3) fatal and/or nonfatal stroke, and (4) fatal CVD. The study‐specific outcome definitions are summarized in Table S2.

The Newcastle‐Ottawa Scale for cohort studies, a tool to assess study quality in terms of cohort and exposure selection, comparability, and outcome assessment, was used to rate the quality of the included studies.^23^ Furthermore, we assessed the quality of the body of evidence on each outcome using the Grading of Recommendations Assessment, Development, and Evaluation tool (https://gdt.gradepro.org) based on study limitations, inconsistency of effect, imprecision, indirectness, and publication bias.^24^ Quality is rated at the following 4 levels: high, moderate, low, and very low.

### Statistical Analysis

Overall summary measures for study‐level characteristics were pooled by a weighted average based on number of study participants.

For the primary analysis, in which we compared ever versus never breastfeeding in parous women, we combined study‐specific hazard ratios for maternal CVD, CHD, stroke, or fatal CVD using random‐effects meta‐analysis. In addition, we applied fixed‐effects meta‐analysis as sensitivity analysis. In case studies provided hazard ratios using different levels of adjustment, we always used the adjustment that accounts for the largest number of variables. When studies only reported separate hazard ratios (1) for different lifetime durations of breastfeeding or (2) for different individual CVD outcomes (eg, CHD, stroke), we combined them using fixed‐effects meta‐analysis to a study‐specific hazard ratio for (1) ever versus never breastfeeding and (2) the composite CVD outcome, respectively.

We investigated the effect of different subgroups on the hazard ratios of the primary analysis using random‐effects meta‐regression.^25^, ^26^ Subgroup analyses were conducted for the variables mean age at study baseline, median duration of follow‐up for cardiovascular events, mean parity, level of adjustment, Newcastle‐Ottawa Scale score (≤6 versus >6), and region (Asia versus other). Moreover, we investigated the influence of single studies on the overall result by conducting leave‐1‐out meta‐analyses, omitting each study in turn.

In the secondary analysis, we report hazard ratios for maternal CVD, CHD, stroke, and fatal CVD for different lifetime durations of breastfeeding. This analysis was based on a dose–response meta‐analysis using a 1‐stage random‐effects dose–response model based on restricted cubic splines with 3 equidistant knots around the range of lifetime duration of breastfeeding (Stata module drmeta).^27^, ^28^ In addition, we tested whether the coefficient of the nonlinear part of the restricted cubic spline was statistically significantly different from 0 and conducted the same analysis assuming a linear trend for all cardiovascular outcomes that had no significant nonlinearity. As lifetime duration of breastfeeding was provided for different time intervals (eg, >6 to 12 months), we computed the midpoint of these intervals and used it for analysis. For studies that did not provide different durations, we analyzed overall mean or median lifetime duration of breastfeeding.

A total of 2 studies^8^, ^10^ reported 95% CIs of hazard ratios using floating absolute risks, and we transformed them back to conventional 95% CIs using methods described elsewhere.^29^ *P* values ≤0.05 were deemed as statistically significant for primary and secondary analyses. After correcting for multiple testing using the Bonferroni method, *P* values ≤0.0021 were deemed as statistically significant for subgroup analyses.^30^ Between‐studies heterogeneity was obtained using the I^2^ statistic.^25^ We used funnel plots to assess potential publication bias and rated their asymmetry with the Egger test,^31^ although they need to be interpreted with caution because of the limited number of studies (<10) for each outcome.

## Results

**Figure 1:**
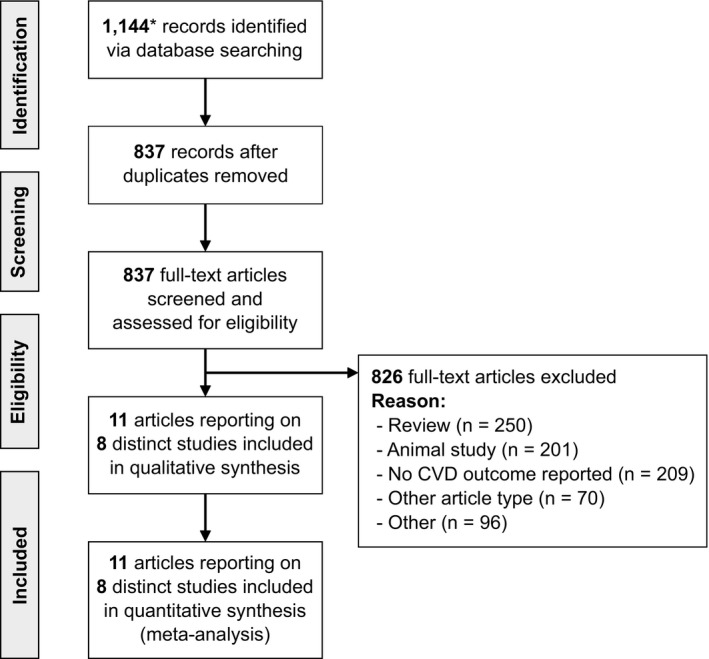
Preferred Reporting Items for Systematic Reviews and Meta‐Analyses flow diagram. CVD indicates cardiovascular disease. *A total of 514 from PubMed and 630 from Web of Science.

The study selection process is demonstrated in Figure 1. Our literature search identified 514 articles via PubMed and 630 articles via Web of Science published until April 16, 2021, which amounted to 837 unique articles after deduplication. We next reviewed the full text of the articles for eligibility and excluded 250 reviews, 201 animal studies, 209 records without maternal cardiovascular outcomes, 70 other article types (eg, case reports or study protocols), and 96 articles for other reasons (eg, articles focusing on cost analyses, individual components of breast milk, or the relationship of breastfeeding with various medical treatments). Finally, 11 articles reporting on 8 distinct studies were eligible for inclusion.

Characteristics of individual studies are provided in the Table.^7^, ^8^, ^9^, ^10^, ^11^, ^12^, ^13^, ^14^, ^15^, ^16^, ^17^ In total, data on 1 192 700 women were included in the analyses. Baseline study years ranged from 1986 to 2009. Weighted mean age was 51.3 years at study entry and 24.6 years at first birth. Women reported a weighted average of 2.3 births. Of the participating women, 982 566 (82%) reported to have ever breastfed. Weighted mean lifetime duration of breastfeeding was 15.6 months. During a weighted median follow‐up duration of 10.3 years, studies reported 54 226 incident maternal CVD events (1 study^15^ did not report the number of incident CVD events), 26 913 incident maternal CHD events, 30 843 incident maternal strokes, and 10 766 maternal fatal CVD events. Studies were generally of high quality, reflected by a weighted overall mean Newcastle‐Ottawa Scale score of 6.5.

**Figure 2:**
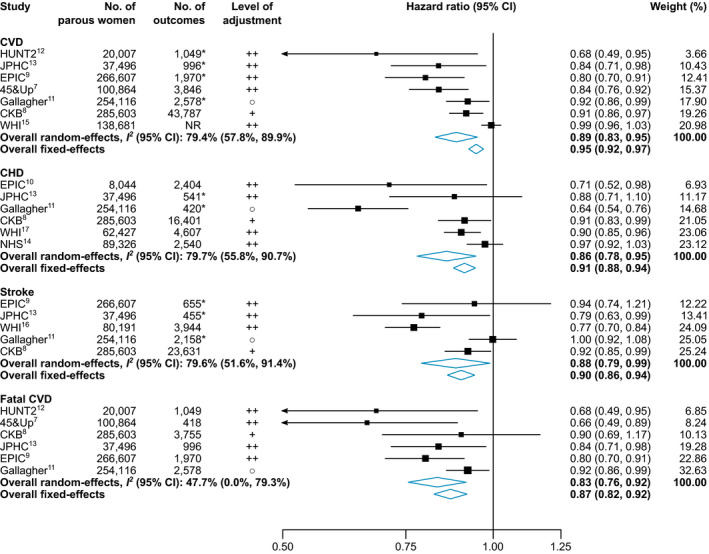
Forest plot for CVD, CHD, stroke, and fatal CVD comparing parous women who ever breastfed vs never breastfed. *Only fatal events included. ○, adjusted for demographics and reproductive factors; +, adjusted for demographics and cardiovascular risk factors; ++, adjusted for demographics, reproductive factors, and cardiovascular risk factors. Full study names are provided in the footnotes of the Table. CHD indicates coronary heart disease; and CVD, cardiovascular disease.

Figure 2 depicts forest plots for each maternal cardiovascular outcome. Hazard ratios comparing women who ever breastfed with those who never did were 0.89 (95% CI, 0.83–0.95; I^2^=79.4% [95% CI, 57.8%–89.9%]) for maternal CVD, 0.86 (95% CI, 0.78–0.95; I^2^=79.7% [95% CI, 55.8%–90.7%]) for maternal CHD, 0.88 (95% CI, 0.79–0.99; I^2^=79.6% [95% CI, 51.6%–91.4%]) for maternal stroke, and 0.83 (95% CI, 0.76–0.92; I^2^=47.7% [95% CI, 0.0%–79.3%]) for maternal fatal CVD using random‐effects meta‐analysis. When using fixed‐effects meta‐analysis, the corresponding pooled hazard ratios were 0.95 (95% CI, 0.92–0.97) for CVD, 0.91 (95% CI, 0.88–0.94) for CHD, 0.90 (95% CI, 0.86–0.94) for stroke, and 0.87 (95% CI, 0.82–0.92) for fatal CVD. Figure S1 demonstrates funnel plots for each cardiovascular outcome. The Egger test was statistically significant for CVD (*P*=0.003), but not for CHD, stroke, and fatal CVD (all *P*>0.05). Of 8 studies, 6 reported hazard ratios comprehensively adjusted for demographic factors, cardiovascular risk factors, and reproductive factors, whereas the other 2 studies (ie, Gallagher et al^11^ and CKB [China Kadoorie Biobank]^8^) reported hazard ratios adjusted for fewer variables. Details on the adjustment of the primary analysis are provided in Table S3. Grading of Recommendations Assessment, Development, and Evaluation ratings for the relevant outcomes together with a summary of findings are reported in Table S4, with the quality of the evidence ranging from very low to moderate. Low evidence was mainly driven by high between‐studies heterogeneity. Figure S2 and Figure S3 depict results of the subgroup analyses. The strengths of associations reported by the studies did not differ according to mean age at baseline, median duration of follow‐up, mean parity, level of adjustment, higher versus lower Newcastle‐Ottawa Scale score, or when comparing studies conducted in Asia versus those conducted in other geographical regions. Leave‐1‐out meta‐analyses omitting each study in turn for all cardiovascular outcomes are presented in Figure S4. Hazard ratios and their 95% CIs remained relatively stable and statistically significant for CVD, CHD, and fatal CVD. However, when omitting the CKB,^8^ the JPHC (Japan Public Health Center–based prospective study)^13^, or the WHI (Women’s Health Initiative)^16^ in the analysis for stroke, the associations were no longer statistically significant. In addition, between‐studies heterogeneity was diminished after removing the WHI study^15^, ^16^ when analyzing the CVD and stroke outcomes and after removing the Gallagher et al^11^ study when analyzing the outcome CHD.

**Figure 3:**
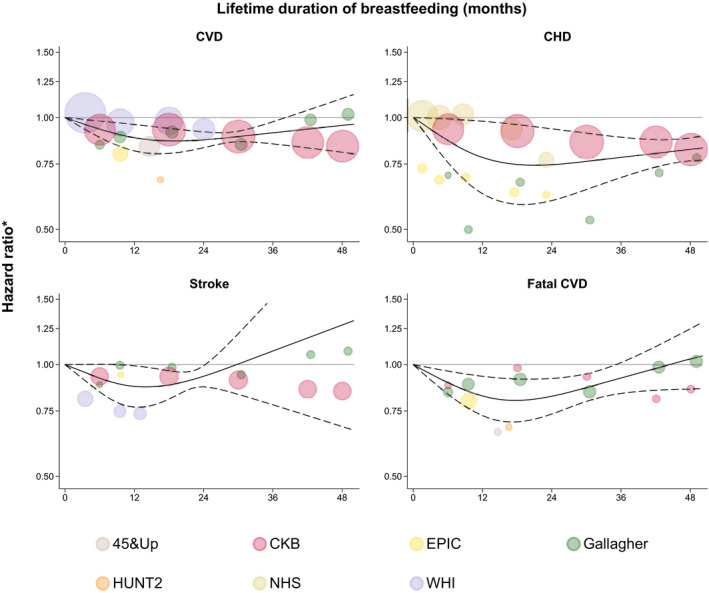
Different lengths of lifetime duration of breastfeeding and incidence of cardiovascular outcomes. *Comparing women with different lifetime durations of breastfeeding with parous women who never breastfed. The solid lines indicate fitted restricted cubic splines and the dashed lines their 95% CIs. The sizes of the circles are proportional to the inverse variance of the hazard ratios. Full study names are provided in the footnotes of the Table. For the purpose of presentation, the graph for stroke has been truncated at 1.50 at the *y* axis. CHD indicates coronary heart disease; and CVD indicates cardiovascular disease.

Results across different lifetime durations of breastfeeding are demonstrated in Figure 3. When modeling the association using restricted cubic splines, relative risks for developing maternal CVD, CHD, stroke, and fatal CVD compared with parous women who never breastfed decreased significantly with lifetime durations of breastfeeding up to ≈12 months. Relative risks appeared to be stable for CVD and fatal CVD between 12 and 24 months of lifetime breastfeeding durations and for CHD between 12 and 48 months, whereas the shapes of associations for longer lifetime durations of breastfeeding were uncertain. When assuming a linear trend for outcomes without a statistically significant nonlinearity in the restricted cubic splines model—that are, CVD, CHD, and stroke—each additional year of breastfeeding resulted in hazard ratios of 0.91 (95% CI, 0.84–0.99; *P*=0.031) for CVD, 0.89 (95% CI, 0.80–1.00; *P*=0.039) for CHD, and 0.91 (95% CI, 0.78–1.06; *P*=0.212) for stroke.

## Discussion

The fact that breastfeeding is associated with cardiovascular risk has been overlooked for a long time. The first article relating lactation to incidence of maternal CVD was published only some years ago, namely in 2008 by Stuebe et al.^14^ Thereafter, investigators examined this association in different studies and populations. We now conducted a systematic literature search and combined results from 8 distinct studies involving >1 million women in a meta‐analysis. We found that parous women who ever breastfed during their lifetime had a reduced risk for developing CVD, CHD, stroke, and fatal CVD compared with parous women who never breastfed. In particular, they had a relative risk reduction of 11% (95% CI, 5% ‐17%) for CVD events, 14% (5% ‐22%) for CHD events, 12% (1% ‐21%) for stroke events, and 17% (8% ‐24%) for fatal CVD events. In addition, our analysis suggests a decreasing risk for maternal CVD, CHD, stroke, and fatal CVD for longer lifetime durations of breastfeeding for up to 12 months.

### Link Between Breastfeeding and Cardiovascular Risk

There exist several theories about the link between breastfeeding and cardiovascular risk. One suggests an involvement of hormones that play important roles during lactation. In particular, prolactin and oxytocin are 2 prominent hormones important in breastfeeding.^32^ Several studies have assessed the effect of prolactin on cardiovascular risk and reported conflicting results.^33^ In contrast, oxytocin has not only been identified to be crucial for ejecting breast milk but also has recently been demonstrated to have several beneficial effects on the cardiovascular system. These include, for instance, blood pressure–lowering effects, vasodilatation, antidiabetic actions, antioxidant effects, inhibition of inflammation, and lowering of fat mass.^34^, ^35^

Another theory relies on the weight loss of women who breastfeed after giving birth. Breastfeeding could facilitate more rapid weight loss in women after delivery. However, previous studies investigating the relationship between lactation and weight change reported conflicting results as described in a systematic review by Neville et al,^36^ although most of the high‐quality studies included in the review demonstrated a direct association between lactation and postpregnancy weight change. Because it is known that elevated weight is a risk factor for future cardiovascular events, it might also be a mediating factor for the association between breastfeeding and reduced cardiovascular risk.

In line with this theory, Stuebe and Rich‐Edwards proposed the hypothesis that lactation may reset maternal metabolism.^37^ This involves the resetting of several metabolic disturbances such as diabetes or hyperlipidemia that are in turn associated with an increased cardiovascular risk. In fact, a variety of studies have demonstrated the reestablishment of glucose and lipid homeostasis after pregnancy and the beneficial effects of lactation on this reconstruction.^37^ Long‐term effects of metabolic dysfunction have also been identified as reflected in a recent systematic review by Pinho‐Gomes et al that included 22 studies and reported a pooled risk ratio of 0.73 (95% CI, 0.65–0.83) for risk of developing type 2 diabetes comparing women who ever lactated with those who never did.^6^ In addition to metabolic risk factors, lactation has been related to other cardiovascular risk factors. For instance, breastfeeding was also associated with a lower risk for hypertension as demonstrated in 2 studies.^38^, ^39^

Studies also showed a relationship between lactation and having subclinical atherosclerosis.^40^, ^41^, ^42^, ^43^ The Coronary Artery Risk Development in Young Adults Study, for example, showed a graded inverse association of the duration of lactation with carotid intima‐media thickness.^41^ Moreover, the Study of Women Across the Nation–Heart Study demonstrated that women who breastfed their children for ≥3 months had a significantly lower risk for aortic calcification and coronary calcification compared with women who did not breastfeed.^43^ All of these studies illustrate possible avenues of how breastfeeding could influence cardiovascular risk factors and thereby have a knock‐on effect on development of clinical cardiovascular events.

### Dose–Response Relationship

The potential existence of an inverse dose–response relationship of breastfeeding duration and cardiovascular risk gained increasing attention during the past years. In 2017, Nguyen et al summarized the association between breastfeeding and maternal CVD in a systematic review,^38^ which was updated in 2020 by Okoth et al.^22^ Both reviews suggested that longer durations of breastfeeding are associated with a reduced risk for maternal CVD. However, it is notable that these reviews only included narrative summaries of the available studies. In the current meta‐analysis, we now quantify the specific association between different lifetime durations of breastfeeding and incidence of cardiovascular outcomes by applying dose–response meta‐analyses. We found a significantly decreased risk for maternal CVD, CHD, stroke, and fatal CVD for lifetime durations of breastfeeding for up to 12 months. After this time, the effect on CHD appeared to reach a plateau between 12 and 48 months. For the other cardiovascular outcomes, there was insufficient data to estimate precisely the shape of association for a longer duration. Therefore, additional studies are needed to provide clarity about the effect of particularly long breastfeeding durations on CVD, stroke, and fatal CVD. Nevertheless, as recommended by the World Health Organization,^1^ children should be exclusively breastfed for the first 6 months of life and will receive an increasing amount of complimentary food afterwards leading to less milk production for longer durations of breastfeeding. This may be a potential explanation for an especially high relevance of lower months of lifetime duration of breastfeeding.

### Dependence on Parity

The average number of children a woman is giving birth to is declining globally. From 1950 to 2019, the global total fertility rate decreased from 4.97 to 2.31.^44^ It is clear that women who have given birth to more children tend to have longer lifetime durations of breastfeeding. To take this into account, Peters et al investigated whether there exists a relationship between duration of breastfeeding per child and cardiovascular risk in the CKB.^8^ Multivariable adjusted analyses suggested an association between longer durations of breastfeeding per child and incidence of cardiovascular outcomes compared with never breastfeeding. Specifically, in parous women who ever breastfed, each additional 6 months of breastfeeding per child were associated with a decreased risk of 4% (95% CI, 2%–5%) for CHD and 3% (2%–4%) for stroke. Furthermore, when restricting their analyses to women with 1 live birth, similar effects were obtained. In line, a longer duration of breastfeeding per child was associated with a reduced risk for CHD in the EPIC‐CVD (European Prospective Investigation Into Cancer and Nutrition–Cardiovascular Disease) study.^10^ An analysis of the Danish National Birth Cohort investigated the duration of breastfeeding for 1 child and found a longer duration of breastfeeding to be associated with risk of CVD, although this was influenced by prepregnancy body mass index.^19^ In the 45 and Up study, no evidence for a dose–response relationship between average duration of breastfeeding per child and CVD hospitalization and mortality was found.^7^ In the current meta‐analysis, mean parity was rather consistent across the included studies and ranged from 2.0 to 3.1 with a weighted mean value of 2.3. Furthermore, the majority of studies took the number of children a woman has given birth to into account by adjusting their analyses for parity or number of live births.

### Clinical Implications

Although the beneficial effects of breastfeeding for children are widely communicated, the positive effects on maternal health are less known. Beside the inverse association of breastfeeding with maternal cardiovascular outcomes shown in our study, breastfeeding is also robustly associated with a reduced risk for maternal type 2 diabetes, ovarian cancer, and breast cancer.^2^ In a survey of 5554 women in the United States, only 38.5% were aware that breastfeeding is associated with a reduced risk for maternal breast cancer.^45^ Moreover, it has been demonstrated that awareness of the World Health Organization breastfeeding recommendations was associated with adhering to them.^46^, ^47^, ^48^ Consequently, there is room for improvement in raising awareness of breastfeeding recommendations and communicating positive effects of lactation not only on children but also on their mothers.

There are only a few conditions that contraindicate breastfeeding.^49^ In the current meta‐analysis, a large proportion of women (82%) reported to have ever breastfed. However, the decision to initiate breastfeeding is affected by a multifaceted variety of factors including, for instance, a women’s work situation, experiences of relatives, and individual‐level factors such as smoking, being overweight, and feeling depressed.^50^ Several interventions have been demonstrated to have a positive effect on breastfeeding initiation and continuation, including education and support by health systems, antenatal and postnatal support to families, and a breastfeeding‐friendly work environment among others.^50^ It is necessary to implement and reinforce such interventions to promote and facilitate breastfeeding.

### Strengths and Limitations

The present study has several strengths. We conducted a comprehensive systematic review of the literature, which—to the best of our knowledge—covers all of the relevant work related to the topic investigated. In addition, our meta‐analysis includes >1 million parous women with several thousand cardiovascular outcomes, which is >4 times more than the previously largest individual study. Furthermore, we applied a consistent definition of the exposure variable with a common reference group (ie, having never breastfed) across studies and compared associations for several distinct cardiovascular end points (CVD, CHD, stroke, and fatal CVD). We also explored for sources of heterogeneity and assessed the dose–response relationships between duration of breastfeeding and cardiovascular outcomes. There were several limitations, which were all inherent. Although we evaluated the potential for publication bias using the Egger test, our results need to be interpreted with caution as tests for publication bias have relatively low power when there are few studies (<10) available for pooling. In addition, significant between‐studies heterogeneity was found for the outcomes CVD, CHD, and stroke, and we were unable to find sources for heterogeneity in our subgroup analyses. Notably, the I^2^ statistics decreased substantially after removing specific studies in leave‐1‐out sensitivity analyses. Other sources of heterogeneity may include clinical, methodological, and statistical aspects of these studies, which are difficult to explore using study‐level data. We did not have access to participant‐level data and therefore could not adopt a uniform approach to statistical adjustment and end point definitions. Access to participant‐level data would have also allowed us to adopt uniform definitions of categories of lifetime durations of breastfeeding, which would be superior to the dose–response meta‐analysis of published study‐specific categories. Moreover, breastfeeding behavior was self‐reported in all included studies and was recalled several years after the breastfeeding period, which may bias our results. However, as previously demonstrated in a study of 374 Norwegian women, the agreement between recalled and recorded breastfeeding duration was very accurate, even after a recall period of 20 years after delivery.^51^ Furthermore, the ability and decision to initiate and continue breastfeeding are influenced by a variety of factors, which may act as potential confounders. Finally, although all but 1 study reported hazard ratios comprehensively adjusted for sociodemographic variables, cardiovascular risk factors, and reproductive factors (for details, see Table S3), we cannot rule out residual confounding attributed to other unadjusted or unmeasured variables and potential underestimation of the cause–effect relationship caused by adjustment for a mediator.

## Conclusions

This meta‐analysis demonstrates a reduced maternal CVD risk in parous women who had breastfed compared with parous women who had never breastfed during their lifetimes.

## Sources of Funding

This work was funded by the Austrian Science Fund (P 32488 and T 1253).

## Disclosures

None.

**Table 1:** Characteristics of Included Studies

Study	Country	Year of baseline	No. of women	Mean age at study entry (y)	Mean parity	Mean age at first birth (y)	No. of women who had ever breastfed (%)	Mean lifetime duration of breastfeeding (mo)	CVD	CHD	Stroke	Fatal CVD	Median follow‐up duration (y)	NOS score
45&Up^7^	Australia	2006–2009	100 864	60.2	2.7	25.0	88 347 (88)	14.6	●	○	○	●	6.1* ^,^ ^†^	7
CKB^8^	China	2004–2008	285 603	50.5	2.0	23.4	277 818 (97)	24.0^‡^	●	●	●	●	8.1	7
EPIC^9^, ^10^	Multinational	1992–2000	266 607	50.3^§^	2.3	24.9	212 041 (80)	9.6	●^||^	×^||^	●^||^	●	12.9*	7
			8044	52.6	2.2	NR	7016 (87)	9.7	○	●	○	○	11.1	7
Gallagher^11^	China	1989–1991	254 116	43.0^‡^	2.1	25.7	214 614 (84)	18.9	●^||^	●^||^	●^||^	●	9.6*	5
HUNT2^12^	Norway	1995–1997	20 007	52.1	2.6	23.4	19 350 (97)	16.5	●^||^	○	○	●	14.5	7
JPHC^13^	Japan	1990–1994	37 496	50.6^§^	2.7	25.1	32 622 (87)	NR	●^||^	●^||^	●^||^	●	20.9*	8
NHS^14^	United States	1986	89 326	40–65^¶^	2.5	NR	56 619 (63)	8.4	○	●	○	○	15.1*	6
WHI^15^, ^16^, ^17^	United States	1993–1998	138 681	63.3	3.1	NR	81 155 (59)	9.4	●	○	○	○	7.9	7
			80 191	63.7	2.9	NR	46 699 (58)	7.4	○	○	●	○	12.6	6
			62 427	63.2^§^	3.1	24.3	36 884 (59)	NR	○	●	○	○	12.0	8
Total		1986–2009	1 192 700	51.3	2.3	24.6	982 566 (82)	15.6					10.3	6.5

If different publications were analyzed for a study, the publication with the largest number of participants was used to compute overall estimates; alternatively, if this publication did not report a variable, the publication that provided this information was used. 45&Up indicates 45 and Up Study; ● = provided; ○ = not provided; × = provided but not used for analysis; CHD, coronary heart disease; CKB, China Kadoorie Biobank; CVD, cardiovascular disease; EPIC, European Prospective Investigation Into Cancer and Nutrition; HUNT2, Nord‐Trøndelag Health Survey 2; JPHC, Japan Public Health Center–based prospective study; NHS, Nurses' Health Study; NOS; Newcastle‐Ottawa Scale; NR, not reported; and WHI, Women’s Health Initiative.

* Mean.

^†^ Mean of 6.1 for nonfatal CVD and 5.7 for fatal CVD.

^‡^ Median.

^§^ Nulliparous women included.

^||^ Fatal only.

^¶^ Minimum–maximum.

## Supporting information

Tables S1–S4
Figures S1–S4
