# Supplementary material for: Breastfeeding Is Associated With a Reduced Maternal Cardiovascular Risk: Systematic Review and Meta‐Analysis Involving Data From 8 Studies and 1 192 700 Parous Women
Source: J Am Heart Assoc. 2022 Jan 11;11(2):e022746. doi: 10.1161/JAHA.121.022746 (PMC9238515; doi:10.1161/JAHA.121.022746)
Supplement: Supplementary file 1 — Tables S1–S4 Figures S1–S4 [file JAH3-11-e022746-s001.pdf]

SUPPLEMENTAL MATERIAL

Table S1. PRISMA checklist

Section/topic	#	Checklist item	Reported on page #
**Title**			
Title	1	Identify the report as a systematic review, meta-analysis, or both.	1
**Abstract**			
Structured summary	2	Provide a structured summary including, as applicable: background; objectives; data sources; study eligibility criteria, participants, and interventions; study appraisal and synthesis methods; results; limitations; conclusions and implications of key findings; systematic review registration number.	2-3
**Introduction**			
Rationale	3	Describe the rationale for the review in the context of what is already known.	4-5
Objectives	4	Provide an explicit statement of questions being addressed with reference to participants, interventions, comparisons, outcomes, and study design (PICOS).	5
**Methods**			
Protocol and registration	5	Indicate if a review protocol exists, if and where it can be accessed (e.g., Web address), and, if available, provide registration information including registration number.	-
Eligibility criteria	6	Specify study characteristics (e.g., PICOS, length of follow-up) and report characteristics (e.g., years considered, language, publication status) used as criteria for eligibility, giving rationale.	5
Information sources	7	Describe all information sources (e.g., databases with dates of coverage, contact with study authors to identify additional studies) in the search and date last searched.	5-6
Search	8	Present full electronic search strategy for at least one database, including any limits used, such that it could be repeated.	5
Study selection	9	State the process for selecting studies (i.e., screening, eligibility, included in systematic review, and, if applicable, included in the meta-analysis).	6
Data collection process	10	Describe method of data extraction from reports (e.g., piloted forms, independently, in duplicate) and any processes for obtaining and confirming data from investigators.	6
Data items	11	List and define all variables for which data were sought (e.g., PICOS, funding sources) and any assumptions and simplifications made.	6
Risk of bias in individual	12	Describe methods used for assessing risk of bias of individual studies (including specification of whether this	6-7

studies		was done at the study or outcome level), and how this information is to be used in any data synthesis.	
Summary measures	13	State the principal summary measures (e.g., risk ratio, difference in means).	7
Synthesis of results	14	Describe the methods of handling data and combining results of studies, if done, including measures of consistency (e.g., I^2) for each meta-analysis.	7-8
Risk of bias across studies	15	Specify any assessment of risk of bias that may affect the cumulative evidence (e.g., publication bias, selective reporting within studies).	8
Additional analyses	16	Describe methods of additional analyses (e.g., sensitivity or subgroup analyses, meta-regression), if done, indicating which were pre-specified.	7-8
**Results**			
Study selection	17	Give numbers of studies screened, assessed for eligibility, and included in the review, with reasons for exclusions at each stage, ideally with a flow diagram.	8-9, Figure 1
Study characteristics	18	For each study, present characteristics for which data were extracted (e.g., study size, PICOS, follow-up period) and provide the citations.	9, Table 1
Risk of bias within studies	19	Present data on risk of bias of each study and, if available, any outcome level assessment (see item 12).	9, Table 1, eTable 3
Results of individual studies	20	For all outcomes considered (benefits or harms), present, for each study: (a) simple summary data for each intervention group (b) effect estimates and confidence intervals, ideally with a forest plot.	Figure 2
Synthesis of results	21	Present results of each meta-analysis done, including confidence intervals and measures of consistency.	9, Figure 2
Risk of bias across studies	22	Present results of any assessment of risk of bias across studies (see Item 15).	9-10, Figure 2,
Additional analysis	23	Give results of additional analyses, if done (e.g., sensitivity or subgroup analyses, meta-regression [see Item 16]).	9-10, eFigure 1, eFigure 2, Figure 3
**Discussion**			
Summary of evidence	24	Summarize the main findings including the strength of evidence for each main outcome; consider their relevance to key groups (e.g., healthcare providers, users, and policy makers).	10-14
Limitations	25	Discuss limitations at study and outcome level (e.g., risk of bias), and at review-level (e.g., incomplete retrieval of identified research, reporting bias).	15
Conclusions	26	Provide a general interpretation of the results in the context of other evidence, and implications for future research.	16
**Funding**			
Funding	27	Describe sources of funding for the systematic review and other support (e.g., supply of data); role of funders for the systematic review.	16

Table S2. Outcome definitions

Study	CVD	CHD	Stroke	fatal CVD
**45&Up**	I20-I25, I61-I67, I69	-	-	I20-I25, I61-I67, I69
**CKB**	I00-I99 (fatal), I20-I25, I60-I69	I20-I25	I60-I69	I00-I99
**EPIC**	I00-I99	I20-I25	I60-I69	I00-I99
**Gallagher**	410-414, 421, 434	410-414	431, 434	410-414, 421, 434
**HUNT2**	I00-I99	-	-	I00-I99
**JPHC**	I20-I52, I60-I69	I20-I52	I60-I69	I20-I52, I60-I69
**NHS**	-	MI or fatal CHD	-	-
**WHI**	CHD, stroke, CHF, angina, PVD, CAD, or coronary revascularization	MI, fatal CHD, CABG, or PTCA	ischemic or hemorrhagic stroke	-

Codes correspond to the International Classification of Diseases (ICD) version 9 or 10. Abbreviations: CABG, coronary artery bypass graft; CAD, coronary artery disease; CHD, coronary heart disease; CHF, chronic heart failure; CVD, cardiovascular disease; MI, myocardial infarction; PTCA, percutaneous transluminal coronary angioplasty. Full study names are provided in the footnote of **Table 1**.

Table S3. Variables in the adjustment of the primary analysis.

Study acronym	45&Up	CKB	EPIC		Gallagher	HUNT2	JPHC	NHS		WHI	
**[Ref]**	[7]	[8]	[9]	[10]	[11]	[12]	[13]	[14]	[16]	[15]	[17]
**Demographics**											
Age	x	x	x	x	x	x	x	x	x	x	x
Country of birth/ethnicity	x	-	-	-	-	-	-	-	x	x	-
Study center/area	-	x	x	x	x	-	-	-	x	-	-
Extension study inclusion	-	-	-	-	-	-	-	-	x	-	-
Socioeconomic status	x	x	x	x	-	x	x	-	x	x	-
Socioeconomic status score	x	-	-	-	-	-	-	-	-	-	-
Income	-	x	-	-	-	-	-	-	-	x	-
Education	x	x	x	x	-	x	-	-	x	x	-
Job status	-	-	-	-	-	-	x	-	-	-	-
Living arrangement	-	-	-	-	-	-	x	-	-	-	-
Marital status	x	-	-	-	-	x	-	-	-	-	-
**Cardiovascular risk factors**											
Body mass index	x	x	x	x	-	-	-	x	x	x	-
Birthweight of subject	-	-	-	-	-	-	-	x	-	-	-
Smoking status/history/duration	x	x	x	x	-	x	x	x	x	x	x
Alcohol intake	x	x	-	-	-	-	x	x	-	-	-
Systolic blood pressure	-	x	-	-	-	-	-	-	-	-	x
History of hypertension/antihypertensive treatment	x	x	-	x	-	-	x	-	-	-	x
History of diabetes/antidiabetic treatment	x	x	-	x	-	-	x	-	-	-	x
Aspirin use	x	-	-	-	-	-	-	x	-	x	-
Total cholesterol	-	-	-	x	-	-	-	-	-	-	-
High-density lipoprotein cholesterol	-	-	-	x	-	-	-	-	-	-	-
Dyslipidemia	-	-	-	-	-	-	-	-	-	-	x
Omega 3 fatty acid use	x	-	-	-	-	-	-	-	-	-	-
Multivitamin use	x	-	-	-	-	-	-	x	x	x	-
Diet	-	-	-	-	-	-	x	x	x	x	-
Physical activity	x	x	x	-	-	x	x	x	x	x	-
Family history of CVD/diabetes/hypertension	x	-	-	-	-	-	-	x	x	x	-
**Reproductive factors**											
Parity	x	-	-	-	-	x	x	x	x	x	-
Number of livebirths	-	-	-	x	x	-	-	-	-	-	-
History/number of stillbirth/s	-	-	-	-	-	-	-	x	-	-	x
Number of miscarriages	-	-	-	-	-	-	-	-	-	-	x
Age at first child	x	-	-	-	-	-	-	-	-	-	x
Age at last child	x	-	-	-	-	-	-	-	-	-	-
Age at menarche	-	-	-	-	-	-	-	-	x	-	-
Age at menopause	-	-	-	-	-	-	-	-	-	x	-
Menopausal status	-	-	x	-	-	-	-	x	-	-	-
Total fertility span	-	-	-	-	-	-	x	-	-	-	-
Hormone intake	x	-	-	-	-	-	x	x	-	-	-
**Level of adjustment**	++	+	++	++	○	++	++	++	++	++	++

○, adjusted for demographics and reproductive factors; +, adjusted for demographics and cardiovascular risk factors; ++, adjusted for demographics, reproductive factors, and cardiovascular risk factors. Abbreviations: CVD, cardiovascular disease. Full study names are provided in the footnote of **Table 1**.

Table S4. GRADE summary of findings.

	CVD	CHD	Stroke	Fatal CVD
**Certainty assessment**				
**No. of studies**	7	6	5	6
**Study design**	observational studies	observational studies	observational studies	observational studies
**Risk of bias**	not serious	not serious	not serious	not serious
**Inconsistency**	very serious (I^2=79.4%)	very serious (I^2=79.7%)	very serious (I^2=79.6%)	not serious
**Indirectness**	not serious	not serious	not serious	not serious
**Imprecision**	not serious	not serious	not serious	not serious
**Other considerations**	publication bias strongly suspected (P_{Egger}=0.003), dose response gradient	dose response gradient	dose response gradient	dose response gradient
**Relative effect (95% CI)**	**HR 0.89** (0.83-0.95)	**HR 0.86** (0.78-0.95)	**HR 0.88** (0.79-0.99)	**HR 0.83** (0.76-0.92)
**Certainty**	⨁◯◯◯ VERY LOW	⨁◯◯◯ VERY LOW	⨁◯◯◯ VERY LOW	⨁⨁⨁◯ MODERATE

Abbreviations: CHD, coronary heart disease; CVD, cardiovascular disease; CI, confidence interval; HR, hazard ratio.

Figure S1. Funnel plots for each cardiovascular outcome.

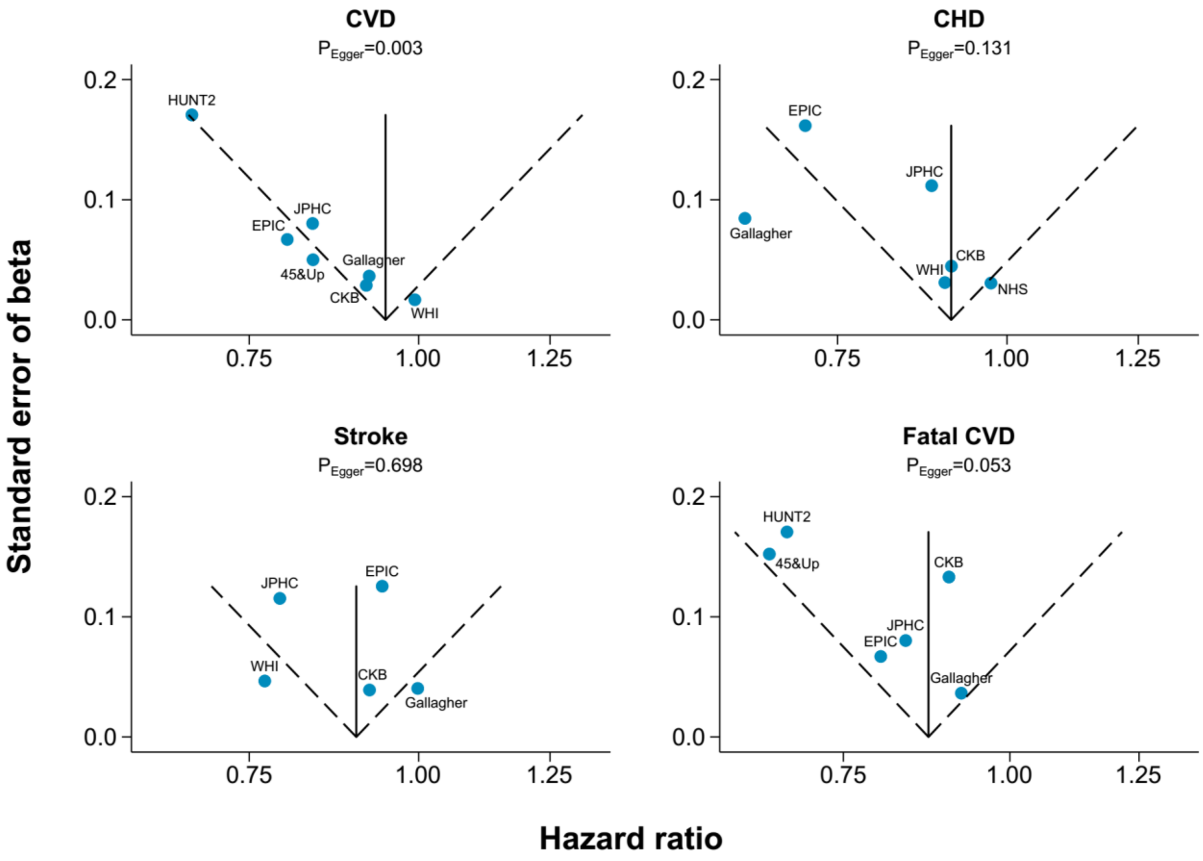

Abbreviations: CHD, coronary heart disease; CVD, cardiovascular disease. Full study names are provided in the footnote of **Table 1**.

Figure S2. Subgroup analyses according to mean age at baseline, median duration of follow-up, and mean parity.

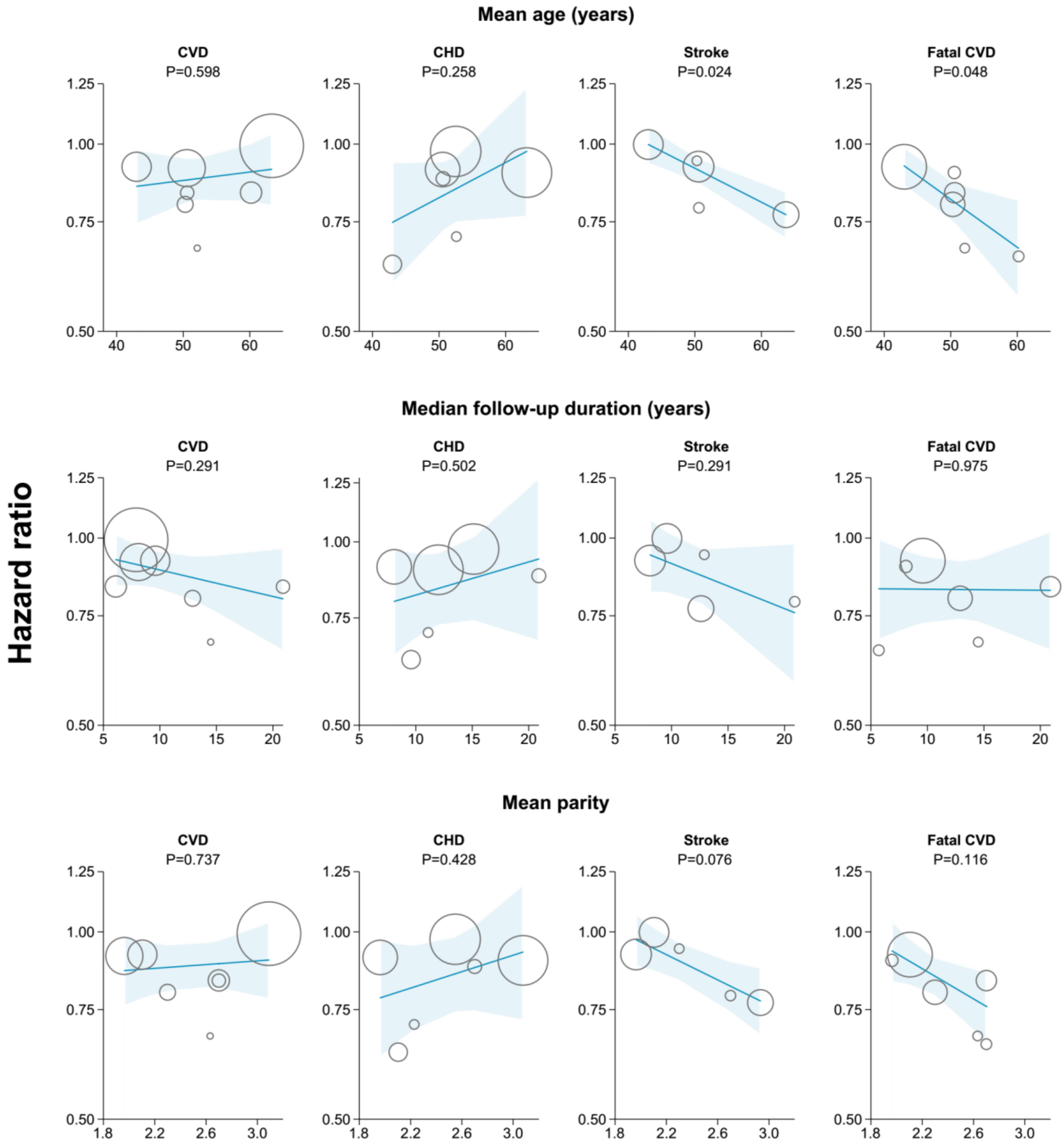

Abbreviations: CHD, coronary heart disease; CVD, cardiovascular disease. Sizes of the circles are proportional to the variance of the effect estimates. Solid lines indicate fitted meta-regression lines and shaded areas their 95% confidence interval. P-values are derived from meta-regression.

Figure S3. Subgroup analyses according to level of adjustment and Newcastle-Ottawa Scale.

Subgroup	No. of studies	No. of parous women	Hazard ratio (95% CI)	P value*
**CVD**				
Adjustment				
○	1	254,116	0.92 (0.86, 0.99)	0.782
+	1	285,603	0.91 (0.86, 0.97)	
++	5	563,655	0.85 (0.75, 0.97)	
NOS				
≤6	1	254,116	0.92 (0.86, 0.99)	0.647
>6	6	849,258	0.87 (0.80, 0.95)	
Region				
Asia	3	577,215	0.91 (0.87, 0.95)	0.697
Other	4	526,159	0.85 (0.73, 0.99)	
**CHD**				
Adjustment				
○	1	254,116	0.64 (0.54, 0.76)	0.110
+	1	285,603	0.91 (0.83, 0.99)	
++	4	197,293	0.92 (0.85, 0.99)	
NOS				
≤6	2	343,442	0.80 (0.53, 1.20)	0.672
>6	4	393,570	0.90 (0.85, 0.94)	
Region				
Asia	3	577,215	0.80 (0.64, 1.01)	0.508
Other	3	159,797	0.92 (0.84, 1.00)	
**Stroke**				
Adjustment				
○	1	254,116	1.00 (0.92, 1.08)	0.141
+	1	285,603	0.92 (0.85, 0.99)	
++	3	384,294	0.79 (0.72, 0.87)	
NOS				
≤6	2	334,307	0.88 (0.68, 1.13)	0.946
>6	3	589,706	0.91 (0.85, 0.97)	
Region				
Asia	3	577,215	0.93 (0.85, 1.03)	0.266
Other	2	346,798	0.82 (0.68, 0.99)	
**Fatal CVD**				
Adjustment				
○	1	254,116	0.92 (0.86, 0.99)	0.167
+	1	285,603	0.90 (0.69, 1.17)	
++	4	424,974	0.79 (0.72, 0.86)	
NOS				
≤6	1	254,116	0.92 (0.86, 0.99)	0.071
>6	5	710,577	0.80 (0.73, 0.87)	
Region				
Asia	3	577,215	0.90 (0.85, 0.96)	0.063
Other	3	387,478	0.76 (0.68, 0.86)	

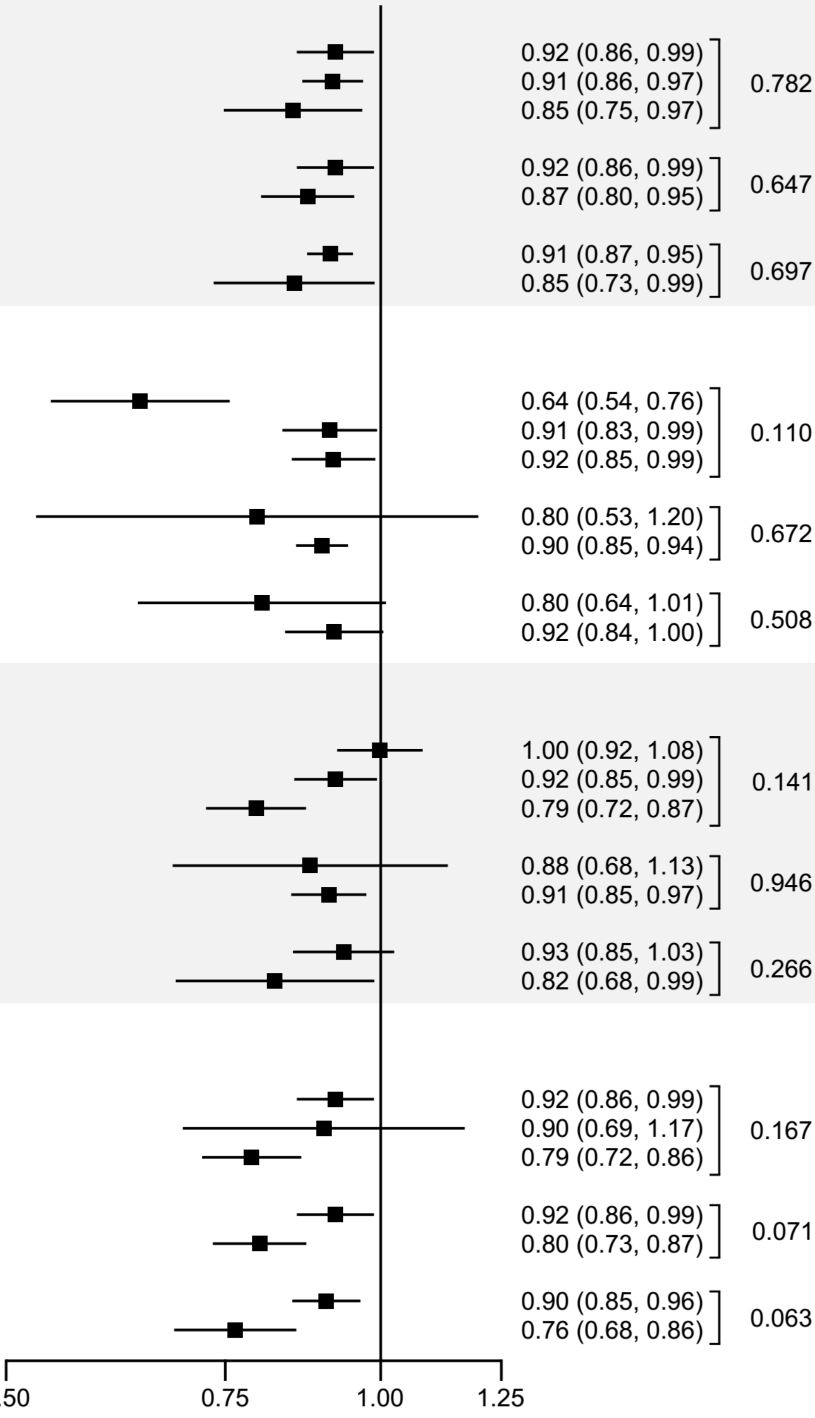

○, adjusted for demographics and reproductive factors; +, adjusted for demographics and cardiovascular risk factors; ++, adjusted for demographics, reproductive factors, and cardiovascular risk factors. *P value for heterogeneity. Abbreviations: CHD, coronary heart disease; CVD, cardiovascular disease; NOS, Newcastle-Ottawa Scale.

Figure S4. Leave-one-out meta-analysis for each cardiovascular outcome.

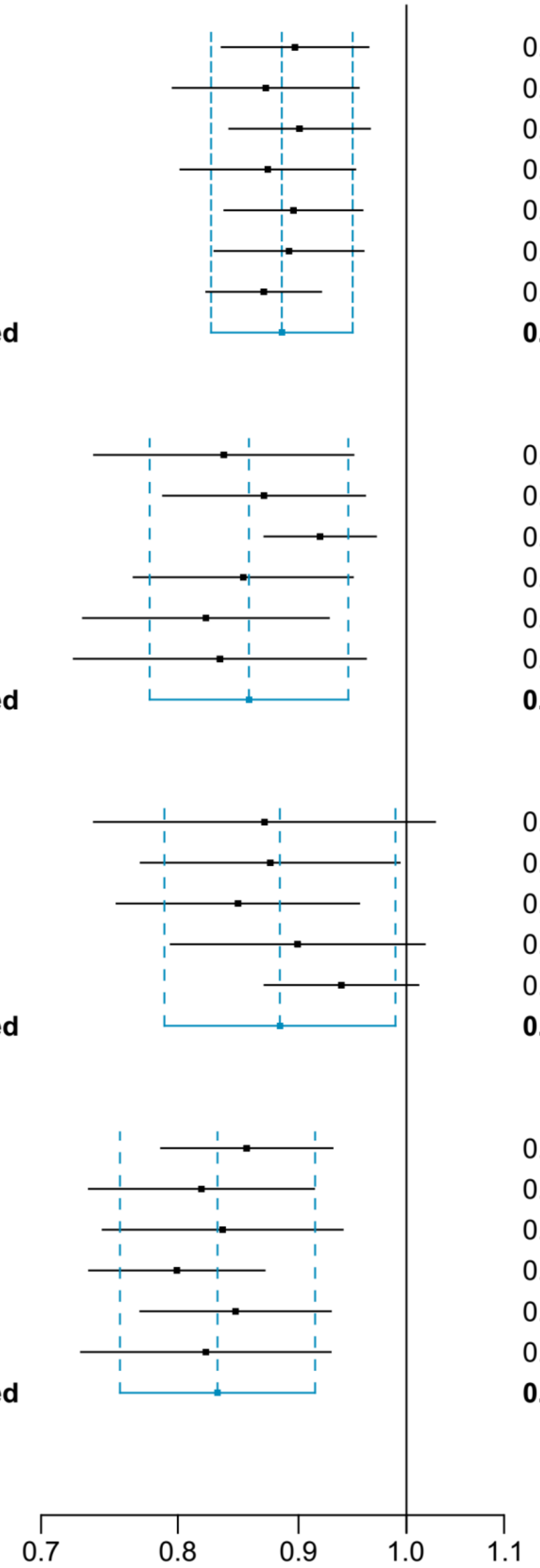

Outcome/omitted study	Hazard ratio (95% CI)	I^2 (95% CI), %
**CVD**		
45&Up	0.90 (0.83, 0.96)	77.9 (51.2, 90.0)
CKB	0.87 (0.80, 0.96)	81.8 (61.3, 91.5)
EPIC	0.90 (0.84, 0.97)	77.9 (51.2, 90.0)
Gallagher	0.87 (0.80, 0.95)	82.4 (62.8, 91.7)
HUNT2	0.90 (0.84, 0.96)	80.3 (57.4, 90.9)
JPHC	0.89 (0.83, 0.96)	81.3 (59.8, 91.3)
WHI	0.87 (0.82, 0.92)	43.9 (0.0, 77.8)
**No study omitted**	**0.89 (0.83, 0.95)**	**79.4 (57.8, 89.9)**
**CHD**		
CKB	0.84 (0.74, 0.95)	83.8 (63.3, 92.8)
EPIC	0.87 (0.79, 0.96)	82.0 (58.5, 92.2)
Gallagher	0.92 (0.87, 0.97)	38.9 (0.0, 77.3)
JPHC	0.85 (0.77, 0.95)	83.7 (63.1, 92.8)
NHS	0.82 (0.73, 0.93)	76.3 (42.1, 90.3)
WHI	0.83 (0.72, 0.96)	83.6 (63.0, 92.8)
**No study omitted**	**0.86 (0.78, 0.95)**	**79.7 (55.8, 90.7)**
**Stroke**		
CKB	0.87 (0.74, 1.03)	84.3 (60.5, 93.7)
EPIC	0.88 (0.77, 0.99)	84.6 (61.5, 93.8)
Gallagher	0.85 (0.75, 0.96)	68.8 (9.9, 89.2)
JPHC	0.90 (0.79, 1.02)	83.6 (58.4, 93.5)
WHI	0.94 (0.87, 1.01)	36.7 (0.0, 78.2)
**No study omitted**	**0.88 (0.79, 0.99)**	**79.6 (51.6, 91.4)**
**Fatal CVD**		
45&Up	0.86 (0.79, 0.93)	35.4 (0.0, 75.8)
CKB	0.82 (0.73, 0.91)	57.9 (0.0, 84.3)
EPIC	0.84 (0.74, 0.94)	48.2 (0.0, 81.0)
Gallagher	0.80 (0.73, 0.87)	0.0 (0.0, 79.2)
HUNT2	0.85 (0.77, 0.93)	46.4 (0.0, 80.3)
JPHC	0.82 (0.73, 0.93)	57.0 (0.0, 84.0)
**No study omitted**	**0.83 (0.76, 0.92)**	**47.7 (0.0, 79.3)**

Abbreviations: CHD, coronary heart disease; CI, confidence interval; CVD, cardiovascular disease. Full study names are provided in the footnote of **Table 1**.
